# Quadruplets

**Published:** 1875-05-15

**Authors:** J. J. Schneck

**Affiliations:** Mt. Carmel, Ill.


### QUADRUPLETS.

NOTES FROM PRACTICE OF J. J. SCHNECK, M.D., MT. CARMEL, ILL.

WAS called at 10 A. M. May 2, 1875, to attend Mrs. Silas Andrus; a woman somewhat below the medium size; sandy complexion; aged thirty-eight years; eighth accouchment; American; and was given the following history:

She had regular menstrual returns until the first of December, 1874, the last three times not being in normal quantity; felt motion for the first time about the first of February, 1875; had felt as well as usual when in the pregnant condition, but thought herself much larger than in previous pregnancies at the corresponding stage.

At 5 A.M., labor pains commenced; at 11 A.M., the first child (a female) was born; and in twenty minutes, a male child was expelled; both vertex presentations. Efficient labor pains now ceased until 1½ P.M., when, after the administration of 1½ drachms of fl. ex. ergot, in three doses, one-half hour apart, and abdominal friction, pains again commenced, and a third child (female) was born; followed by a male child in fifteen minutes; both podalic presentations.

In a few moments the placenta was passed; this was of an oblong shape, and attached to it were four umbilical cords, in as many separate sacs, which were variously adherent to one another as they approached the placental surface. The children are all of about the same size and weight, weighing near two pounds each, and have the appearance of having reached between the seventh and eighth month of fœtal life. The first child born lived twenty-four hours; the other three died within thirty minutes of their respective births.

Neither of the parents have any near relatives that have had plural births.

